# Chronic suppurative lung disease and bronchiectasis in children, adolescents and adults in Australia and New Zealand: TSANZ position statement summary

**DOI:** 10.5694/mja2.52160

**Published:** 2023-11-10

**Authors:** Keith Grimwood, Emma Kennedy, Maree Toombs, Paul J Torzillo, Anne B Chang

**Affiliations:** ^1^ Griffith University Gold Coast QLD; ^2^ Gold Coast Hospital and Health Service Gold Coast QLD; ^3^ Flinders University Darwin NT; ^4^ University of Sydney Sydney NSW; ^5^ Royal Prince Alfred Medical Centre Sydney NSW; ^6^ Institute of Health and Biomedical Innovation, Centre for Children's Health Research Queensland University of Technology Brisbane QLD; ^7^ Queensland Children's Hospital Brisbane QLD

**Keywords:** Bronchial diseases, Bronchitis, Respiratory tract infections

Bronchiectasis is characterised by chronic cough and sputum production, recurrent respiratory infections, and computed tomography (CT) evidence of bronchial dilatation.^1^ Globally, bronchiectasis is the third most common chronic respiratory disorder after asthma and chronic obstructive pulmonary disease.^2^ In Australia, bronchiectasis is most prevalent in Indigenous populations from remote northern communities,^3^ where an estimated 1470 per 100 000 children aged less than 14 years^1^, ^3^ and up to 23% of Indigenous adults undergoing chest CT scans have radiographic evidence of bronchiectasis.^4^ Indigenous Australians with bronchiectasis die about 20 years earlier than non‐Indigenous adults with bronchiectasis, who, in turn, die ten years earlier than other Australians.^5^

Nevertheless, bronchiectasis remains under‐recognised and undertreated. An Australian cohort study reported that over 60% of referred adults with bronchiectasis had symptoms dating from childhood and a worse prognosis than those with adult‐onset disease.^6^ In contrast, bronchiectasis in children is potentially reversible if diagnosed early and treated optimally.^7^, ^8^ Guidelines, therefore, aim for early diagnosis and treatment to reverse mild bronchiectasis in children, thereby reducing future adult bronchiectasis while seeking to preserve lung function and quality of life in others with established disease.^9^, ^10^

## Thoracic Society of Australia and New Zealand position statement

The 2015 Thoracic Society of Australia and New Zealand (TSANZ) guidelines^11^ were replaced recently by an updated TSANZ Position Statement.^12^ As before, the 2023 TSANZ Statement^12^ (Box) seeks to increase awareness of bronchiectasis in health professionals and employs the best available evidence to support its recommendations on diagnosis, underlying causes, airway clearance, controlling infection and preventing complications. Here, we discuss within the Australian context the main differences between these two publications,^11^, ^12^ followed by implementation and future research directions.

Box 1 **Summary of the Thoracic Society of Australia and New Zealand (TSANZ) statement^12^**

**Table:** 

	Recommendations
When to consider bronchiectasis	**Child/adolescent:** recurrent (> 3 episodes per year) or chronic (> 4 weeks’ duration) wet/productive cough, chronic wet/productive cough not responding to 4 weeks of antibiotics, recurrent episodes of pneumonia (≥ 3 episodes ever), severe asthma (with chronic wet/productive cough), and/or persistent lung parenchymal abnormalities on chest x‐ray (> 6 weeks) **Adults:** chronic cough, sputum production and recurrent respiratory exacerbations, suggestive of the bronchiectasis phenotype, in patients with severe underlying asthma or COPD
How to confirm the diagnosis	**Child/adolescent:** seek specialist advice first and use paediatric‐specific radiographic diagnostic criteria **All age groups:** MDCT scans with HRCT reconstruction
What investigations to perform	Identify underlying causes; severity; comorbid conditions, including treatable traits; and lower airway microbiology Minimum baseline investigations: ‣ **Child/adolescent:** full blood count, major Ig classes (G, A, M and E), sweat test, airway cultures, spirometry if aged > 6 years ‣ **Adults:** full blood count, major Ig classes (G, A, M and E), airway cultures, spirometry, *Aspergillus*‐specific IgE if total IgE is elevated Additional investigations guided by history/examination findings, severity, and identifiable comorbid conditions Severity determined by clinical, spirometric and radiographic findings (eg, bronchiectasis severity index score for non‐Indigenous adults)
How to manage	
General principles	Preserve lung function, halt disease progression, optimise symptom control and QoL, minimise frequency and severity of exacerbations, prevent complications **Child/adolescents:** in addition, optimise lung growth and possibly reverse structural lung injury
Antibiotics	Eradication of newly detected *Pseudomonas aeruginosa* Treatment of respiratory exacerbations Long term therapy to reduce exacerbation frequency in patients with the frequent exacerbator phenotype (≥ 3 episodes per year)
Airway management	
‣ Mucolytics	rhDNAse is contraindicated, and other agents (eg, hypertonic saline, mannitol) should not be used routinely
‣ Airway clearance	Airway clearance should be individualised and reviewed regularly by a respiratory physiotherapist
‣ Bronchodilators	They should not be prescribed routinely, but instead on an individual basis (eg, before administering mucolytics, inhaled antibiotics or airway clearance techniques)
‣ Corticosteroids	They should only be used if there is an established coexisting diagnosis of asthma and/or eosinophilic airway inflammation
Exercise/rehabilitation	Regular exercise is recommended for everyone, including with pulmonary rehabilitation in adults with frequent exacerbations and/or reduced exercise tolerance
General wellbeing	Optimise nutrition; avoid tobacco cigarette and electronic cigarette use and other aerotoxicants; keep up to date with vaccinations according to the national immunisation schedules, including influenza, COVID‐19 and pneumococcal vaccinations
Monitoring	Patients should be reviewed at least every 6–12 months by an interdisciplinary specialist respiratory service that monitors general wellbeing, disease severity, spirometry, and airway cultures; manages treatable traits, complications and comorbid conditions; and develops individualised self‐treatment plans
Surgery	Surgery is performed rarely, but, when considered, the patient requires assessment by an expert interdisciplinary team and is undertaken in a facility able to provide the necessary pre‐ and post‐operative care
Health service equity	Given the high prevalence of bronchiectasis in Indigenous people, every effort should be made to ensure that those affected receive an early diagnosis and best practice treatment — this also extends to those living in underserviced communities and in rural and remote regions of Australia
Transitional care	Developmentally appropriate and planned transition from paediatric to adult health services, involving interdisciplinary teams from both services, should be provided

COPD = chronic obstructive pulmonary disease; COVID‐19 = coronavirus disease 2019; HRCT = high resolution computed tomography; Ig = immunoglobulin; MDCT = multidetector computed tomography; QoL = quality of life; rhDNAse = recombinant human deoxyribonuclease.

The TSANZ 2023 Position Statement^12^ was formulated by an interdisciplinary panel of health professionals caring for patients with bronchiectasis. The 2023 version^12^ is more inclusive than the 2015 Guideline,^11^ with panel members comprising nine medical practitioners, three physiotherapists, a respiratory nurse, an Indigenous academic and a health consumer representative. Several panel members had additional expertise that included infectious diseases and Indigenous and remote and rural health.

For the 2023 update,^12^ each of the 33 statements was allocated to a panel pair (each with paediatric and adult expertise). Systematic reviews for each statement were undertaken and an updated search was performed using dates from the most recent international guidelines from the British Thoracic Society^9^ and the European Respiratory Society^10^ as a baseline. The statements were then reviewed and discussed by the whole panel before circulation as part of a Delphi process to 47 health professionals with expertise in bronchiectasis (19 were surveyed in the 2015 guideline),^11^ of whom 31 responded, and a final series of statements was generated.

Overall, of the 33 individual statements, one is new, 28 are modified, and four unchanged from the 2015 Guideline.^11^ The new and modified statements are often fuller and more detailed than presented in the 2015 Guideline,^11^ and are accompanied by three boxes, a table and a figure.^12^ There is a box outlining the clinical features of bronchiectasis, including trigger points for referring children and adolescents to paediatric respiratory specialist services. Another box describes the minimum panel of investigations to perform for determining underlying aetiology, disease severity, comorbid conditions, and treatable traits. This box also describes circumstances when more extended investigations are warranted and the types of additional tests are listed. The last box presents the main objectives of optimising management for patients with bronchiectasis. The Statement's table provides an antibiotic selection guide for the empirical and directed therapy of acute respiratory exacerbations. Meanwhile, the figure is adapted from the European Respiratory Society guidelines^10^ and suggests a series of pathways for attempting to eradicate newly detected *Pseudomonas aeruginosa* in respiratory secretions. The 2023 Position Statement has an overarching technical report available in appendix 1 of the online supplement.^12^

The possibility of mild bronchiectasis being reversible in children if identified early and treated optimally reinforces the importance of recognising the clinical features of bronchiectasis and using paediatric radiographic diagnostic criteria when interpreting chest CT scans. However, making the diagnosis is the beginning, and not the end, of the diagnostic journey. As bronchiectasis is a heterogenous disorder, a search for an underlying cause and comorbid conditions should be initiated. This includes recognising treatable traits — that is, therapeutic targets identified by phenotypes (observable clinical characteristics) or endotypes (underlying pathobiological pathways associated with outcomes and validated biomarkers) — while determining the extent and severity of the bronchiectasis remains important. The 2023 Statement^12^ does this in greater detail than the 2015 Guidelines.^11^ It lists the possible causes of bronchiectasis and includes clinical features that should trigger additional investigations. Furthermore, as paediatric and adult patients with bronchiectasis can have different aetiologies and comorbid conditions, the baseline and extended investigations are split between these two age groups.

In bronchiectasis, *P. aeruginosa* is associated with worse clinical outcomes,^1^, ^7^ and, consistent with other international guidelines,^9^, ^10^, ^13^ the 2023 Statement^12^ now states that eradication therapy for newly detected *P. aeruginosa* in respiratory secretions should be offered, rather than considered, and provides advice in the figure on how this might be achieved. This was based upon four before and after (two prospective) studies in adults reporting improved quality of life and reduced exacerbations and hospitalisations following eradication, and two systematic reviews of randomised controlled trials of inhaled antibiotics in adults with bronchiectasis and chronic *P. aeruginosa* infection, where eradication was associated with reduced exacerbations but was a secondary outcome.^12^

Although the optimal duration of antibiotic treatment of exacerbations is unknown, a single, double‐blind randomised controlled trial in children and adolescents with non‐severe (non‐hospitalised) exacerbations found that oral amoxycillin–clavulanate was superior to placebo at achieving symptom resolution and shortening symptom duration after a 14‐day treatment.^14^ Accordingly, the 2023 Statement^12^ has recommended antibiotics be continued for at least 14 days, rather than the ten days stated in the 2015 Guidelines.^11^ The goal of long term antibiotics (> 2 months) is to reduce bacterial load and/or airway inflammation when eradication is not possible. The recommendation for using long term oral macrolides has been simplified (considered in those with the frequent exacerbator phenotype [ie, ≥ 3 exacerbations per year], which predicts mortality, hospitalisation, and reduced quality of life)^15^, ^16^ and should be prescribed for at least a six‐month trial with regular review for clinical benefit. In addition, more advice is provided on risk factors and monitoring for rare but severe adverse effects from oral macrolides. However, the advice that inhaled antibiotics should not be prescribed routinely remains unchanged, although they can be considered in adults chronically infected with *P. aeruginosa* and frequent exacerbations when oral macrolides are either contraindicated, not tolerated or fail to reduce exacerbation frequency.

In other statements in the 2023 publication,^12^ electronic cigarettes are now included in products to be avoided, the indications for surgery, pre‐operative assessment and where this should be performed are presented in greater detail, as are statements concerning equity of access to specialist health services, which could mean telehealth for rural and remote regions. Finally, the last and newest statement recommends planned, clearly documented and developmentally appropriate transition from paediatric to adult services, involving interdisciplinary members of both health teams. It seeks to avoid the lack of engagement and adverse health outcomes when transition arrangements are absent.^17^

## Implementation

Although endorsed statements and guidelines are produced, these are not always followed because of insufficient evidence to support their recommendations, conflicting data or a lack of awareness from inadequate dissemination.^18^ To help address the latter, a series of webinars is planned, and TSANZ/Australian Lung Foundation health professional and patient checklists informed by the 2023 Statement^12^ have been developed for distribution. A clinician‐ and patient‐focused consensus quality standards document for managing bronchiectasis derived from the 2023 Position Statement recommendations^12^ is planned, using similar methodology employed by the European Respiratory Society Children's Bronchiectasis Education, Advocacy and Research Network (www.improveBE.org).^19^ As an initial step, the quality standards aim to further improve the health care of patients with bronchiectasis by being an advocacy tool for patients and clinicians and for monitoring health service performance.^20^

## Future directions

Despite the growing evidence base for bronchiectasis, large knowledge gaps remain and no licensed therapies exist. Consequently, guidelines often rely upon expert opinion and need regular updating. For example, a report has just been published of long term azithromycin use being most effective at reducing exacerbations between 17 and 62 weeks after its initiation in children with bronchiectasis,^21^ while a small retrospective case series in children with primary ciliary dyskinesia (19 had bronchiectasis) found that *P. aeruginosa* eradication therapy was successful in 30 of 31 infected children and sustained for at least one year in 19, but follow‐up was insufficient to determine clinical benefit.^22^

The highly heterogenous nature of bronchiectasis is a major clinical and research challenge. This requires grouping patients by their shared pheno‐endotypes using clinical, radiographic, spirometric and multiomics‐derived criteria and validated biomarkers to allow treatable traits (pulmonary, aetiology, extrapulmonary, behaviour and lifestyle) to be identified and managed, as well as improving clinimetric properties in registry‐based studies,^16^ and having well balanced arms in high quality, adequately powered randomised controlled trials.^23^ Additional strategies addressing heterogeneity require standardising definitions (eg, exacerbations)^24^ and adopting patient‐focused core outcome sets (an agreed minimum set of critically important outcomes) to improve trial comparisons, meta‐analyses and guidelines.^25^

Finally, better engagement and investment are also required in allocating resources to culturally and linguistically diverse groups, where disease burden is highest and outcomes poorest.^3^, ^5^ Future statements should depend on studies that ensured the right patient populations received the right interventions and measured the right endpoints.^25^

## Open access

Open access publishing facilitated by Griffith University, as part of the Wiley ‐ Griffith University agreement via the Council of Australian University Librarians.

## Competing interests

No relevant disclosures.

## Provenance

Not commissioned; externally peer reviewed.
